# Counting to two: how phages decide between lysis and lysogeny

**DOI:** 10.64898/2026.05.14.725151

**Published:** 2026-05-17

**Authors:** Janni Harju, Ghita Guessous, Zemer Gitai, Ned Wingreen

**Affiliations:** 1 Joseph Henry Laboratories of Physics, Princeton University, Princeton, NJ, USA.; 2 Department of Molecular Biology, Princeton University, Princeton, NJ 08544; 3 Lewis-Sigler Institute for Integrative Genomics, Princeton University, Princeton, NJ 08544*

## Abstract

Upon infecting a bacterium, temperate phages must decide between killing the cell to reproduce (lysis) or entering a symbiotic lifestyle (lysogeny). This choice is often informed by the cell’s state, as well as the number of infecting phage particles (MOI). Since phage gene copy numbers scale identically with MOI, an MOI-dependent decision requires a fast-acting asymmetry between the lytic and lysogenic pathways. We introduce a minimal model suggesting that only a handful of coupling mechanisms can produce such an asymmetry; for instance via a host protease, kinase, or RNase acting on one pathway. By distilling complex regulatory networks to their essential components, our model clarifies the logic of lysis-lysogeny decision mechanisms across phage species.

## I. INTRODUCTION

Temperate phages are bacterial viruses that must decide between two alternate life-paths upon entering a host. This decision is generally informed by the number of phage particles infecting the host: if the multiplicity of infection (MOI) is one, the phage typically replicates and then lyses the bacterium, releasing a burst of progeny. However, at higher MOIs, which indicate a higher ratio of phage particles to susceptible hosts in the local environment, the phage can instead integrate its genome into the bacterial cell, forming a lysogen [1].

The lysis-lysogeny decision mechanism of the *Escherichia coli* phage *λ* has been extensively studied for over six decades [2, 3]. Early works based on mutant assays identified key genes required for each infection pathway. High levels of the *λ* protein CII promote both integration and expression of CI, a repressor that maintains the lysogenic state. At low MOIs, this pathway is suppressed by degradation of CII by the essential host protease FtsH [4, 5].

Interestingly, more recent studies have identified novel lysis-lysogeny decision mechanisms that are also coupled to host FtsH activity [6, 7]. Both these and the *λ* decision pathway have been successfully modeled *in silico* [7–10]. Although these models can reproduce the MOI dependence of post-infection dynamics, their complexity makes it difficult to extract general principles that could underpin lysis-lysogeny decisions across species. Furthermore, questions remain about how MOI-independent early gene expression dynamics can give rise to MOI-dependent lysis-lysogeny outcomes [10].

Here, we introduce a coarse-grained model that lays out minimal requirements for an MOI-dependent lysislysogeny switch. The model requires a mechanism that controls protein accumulation dynamics on relevant, short timescales and in a targeted manner. Motivated by the established role of FtsH in known systems, we consider protease-mediated degradation of lysogeny-inducing proteins as an illustrative example mechanism. Our model suggests that saturation of protease activity, thought to be relevant for *λ* [2, 10], is not necessary for a functional lysis-lysogeny decision mechanism. Our generalized framework predicts how lysis-lysogeny decisions shift with host state and helps identify the most consequential interactions in such decision pathways.

## II. RESULTS

**FIG. 1.**
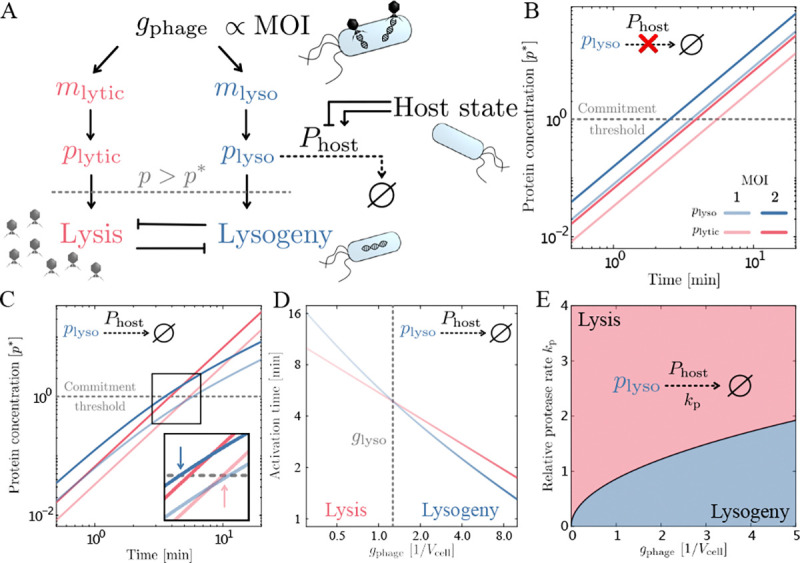
Minimal model for lysis-lysogeny decisions. (A) Illustration of the model. mRNAs for both a lysis- and a lysogeny-promoting protein are transcribed at rates proportional to the multiplicity of infection (MOI). Once either protein concentration, $p_{\text{lyt}}$ or $p_{\text{lyso}}$, reaches a commitment threshold, it triggers its own pathway as well as repression of the alternative pathway. An asymmetry is introduced by host protease-mediated degradation of the lysogeny-promoting protein. (B) Protein dynamics in the absence of host protease-mediated degradation; on short timescales, both protein levels increase quadratically in time, for all MOIs. Parameters were set following [10]. (C) Protein dynamics with host protease-mediated degradation of the lysogeny-promoting protein at rate $k_{\text{p}}$. At timescales $t\sim 1/k_{\text{p}}$, $p_{\text{lyso}}$ transitions to a linear scaling with time. Inset shows that the two protein curves now cross, and a change in the MOI can change which protein reaches the threshold level first. (D) Scalings of the times to reach commitment threshold with respect to the phage genome concentration $g_{\text{phage}}$ within the cell. Above $g_{\text{lyso}}$, the outcome is lysogeny. At large $g_{\text{phage}}$, the threshold is reached at $t<1/k_{\text{p}}$. Concentration units are in phage genomes per cell volume $V_{\text{cell}}$. (E) Phase diagram of the model. Black curve represents $g_{\text{lyso}}\left(k_{\text{p}}\right)$. In the lytic regime, degradation of $p_{\text{lyso}}$ is fast enough compared to transcription to prevent lysogeny.

To construct our model, we assume that the lysislysogeny decision is based on the relative dynamics of two competing pathways (Fig. 1A). At time t=0, the concentration of phage genomes $g_{\text{phage}}$ inside the bacterial host is proportional to the MOI. The phage genome encodes a lysis-promoting and a lysogeny-promoting protein, whose corresponding mRNA and protein concentrations are denoted by $m_{\text{lyt}}/m_{\text{lyso}}$ and $p_{\text{lyt}}/p_{\text{lyso}}$. We assume that each protein has a designated decision threshold concentration $p_{\text{i}}^{*}$. If a protein reaches this level, it activates its own pathway and represses the alternate one. The simplest dynamics for these components consist of transcription, translation, as well as protein and mRNA degradation and dilution (see Methods A). However, since the lysislysogeny decision of λ occurs within the first ∼ 5 minutes post-infection, not all of these processes act on relevant time-scales. Degradation rates for *λ* transcripts are estimated at 1*/*10 min^−1^ [10]. Protein dilution and degradation rates in bacteria are generally on the order of 1*/*20 min^−1^ [11]. Hence, on timescales t≲5 min, the protein and mRNA dynamics are well approximated by

(1) $$\frac{dm_{\text{i}}}{dt}\approx k_{\text{i}}^{\text{tx}}g_{\text{phage}},$$

(2) $$\frac{dp_{\text{i}}}{dt}\approx k_{\text{i}}^{\text{tr}}m_{\text{i}}.$$

Here, i is the index lyt or lyso, and $k_{\text{i}}^{\text{tx}}$ and $k_{\text{i}}^{\text{tr}}$ are the transcription and translation rates. mRNA concentrations therefore increase approximately linearly in time $\left(m_{\text{i}}\approx k_{\text{i}}^{\text{tx}}g_{\text{phage}}t\right)$, and protein levels increase quadratically $\left(p_{\text{i}}\approx k_{\text{i}}^{\text{tr}}k_{\text{i}}^{\text{tx}}g_{\text{phage}}t^{2}/2\right)$.

To visualize the effects of MOI changes on phage protein dynamics, we plot $p_{\text{i}}(t)$ on logarithmic axes, where the slope of each curve is set by the exponent of t; here 2 (Fig. 1B). The vertical separation between the two curves is set by the ratio of products of transcription and translation rates of the two mRNA species. Upon a doubling of the MOI, total transcription rates double, and both curves shift upwards by equal amounts. The order of the parallel curves does not change with MOI, and hence the same protein always reaches the decision threshold first. An MOI-dependent decision mechanism thus requires an asymmetry beyond different transcription and translation rates.

This additional mechanism should specifically target one pathway on timescales < 5 minutes. One possibility would be a nonlinearity in Eqs. (1) or (2), corresponding to feedback between mRNA/protein levels and transcription/translation rates. Alternatively, either pathway might couple to host- or phage-encoded machinery. Any phage-encoded machinery would first have to be transcribed and translated. Since slower post-infection dynamics would give the host more time to mount an immune response, coupling to host-encoded machinery is expected to be favored. Moreover, by basing decisions on host-encoded factors, phage can potentially gain information about their host’s state (see Discussion).

As a biologically motivated example, we consider degradation of the lysogeny-inducing protein by a host protease, e.g. FtsH in *E. coli*. Bacterial proteases are highly specific, and can degrade proteins on timescales of ∼ 2 min [5]. We hence introduce a protease degradation term:

(3) $$\frac{dp_{\text{lyso}}}{dt}=k_{\text{lyso}}^{\text{tr}}m_{\text{lyso}}-k_{\text{p}}p_{\text{lyso}},$$

where $k_{\text{p}}$ is the protease degradation rate. At times $t<1/k_{\text{p}}$, $p_{\text{lyso}}$ still increases quadratically. However, for $t>1/k_{\text{p}}$, the scaling of $p_{\text{lyso}}$ becomes linear (Fig. 1C). This implies that the concentration curves $p_{\text{i}}(t)$ can now intersect. Critically, an increase in the MOI, e.g. from 1 to 2, can now move the crossing point of the two curves across the $p=p^{*}$ line. As a result, a different protein – lytic or lysogenic – can reach the decision threshold first (Fig. 1C inset) while the other protein’s concentration is still ∼ 20% below its decision threshold (see Methods B). Intuitively, at larger MOIs, transcription of $m_{\text{lyso}}$ is sufficiently fast to outpace protease-mediated degradation, thus leading to lysogeny.

Within our model, the decision switches at a genome concentration where $p_{\text{lyt}}$ and $p_{\text{lyso}}$ reach their target levels simultaneously (Fig. 1D). Since the crossing point occurs at time $t_{\text{x}}\sim 1/k_{\text{p}}$ (see Methods C), setting $p_{\text{lyt}}\left(1/k_{\text{p}}\right)\sim p_{\text{lyt}}^{*}$, we find a scaling prediction for the minimum concentration for lysogeny:

(4) $$g_{\text{lyso}}\sim \frac{k_{\text{p}}^{2}}{k^{\text{tx}}k^{\mathrm{tr}}}.$$

Here, $k^{\text{tx}}$ and $k^{\text{tr}}$ are the baseline transcription and translation rates of the phage genes, and a constant prefactor is set by the ratios of $k_{\text{i}}^{\text{tx}}$, $k_{\text{i}}^{\text{trr}}$, and $p_{\text{i}}^{*}$ (see Methods D). Eq. (4) shows that a 30% decrease in the protease degradation rate could shift the MOI at which lysogeny occurs from two to one, effectively preventing lysis (Fig. 1E).

## III. DISCUSSION

The choice between lysis and lysogeny is an informed decision made by genetic systems of remarkable simplicity. We propose that the essential interactions underpinning these decision mechanisms can be identified from the temporal scaling behavior of their key components. This approach illustrates how MOI-dependent decisions can arise despite identical early gene transcription rates across MOIs. [2, 10].

A key aspect of our model is that since temperate phages commit to either pathway within ∼ 5 min following infection, only processes that occur at rates faster than these timescales can influence the decision. We considered protease-mediated degradation of the lysogeny-inducing protein as an example, but our model also points toward alternative mechanisms. For example, enhanced degradation of the lysogeny-promoting mRNA species would cause $m_{\text{lyso}}$ to reach a steady state soon after infection, and consequently, $p_{\text{lyso}}$ would again increase linearly with time. Alternatively, if post-translational modification, e.g. phosphorylation, was required to activate the lytic protein, the concentration of activated lytic protein would increase with $t^{3}$, again resulting in a crossing of the protein concentration curves.

In general, phages may have evolved to use multiple of these “building blocks” as the basis for their MOI-based decision-making. In phage λ, in addition to FtsH-mediated degradation of CII, RNase III destabilizes *cII* transcripts [3]. Nonlinearities in transcription, translation, or degradation rates could also affect decisions. For instance, although our model shows that saturation of host protease activity is not necessary for MOI-dependent decisions, it could nevertheless help stabilize lysogeny-promoting proteins (see Methods E). This would be consistent with the λ protein CIII stabilizing CII by also being targeted by FtsH [12].

Beyond MOI, phages can also base their decisions on host state. For example, a stress-induced transcription factor could directly couple either pathway to host conditions, without introducing an MOI-dependence. More subtly, an MOI-dependent mechanism, such as coupling to a host protease, could also inform phages of host state (Eq. (4)). Responding to new conditions — including starvation, heat/osmotic/membrane stress, as well as quorum-sensing signals — requires bacteria to extensively restructure their proteomes, thus reducing protease availability; phages can hence use proteases as a general indicator of host health, favoring lysogeny under stressful conditions [13–15].

Our model suggests that the number of possible mechanisms for lysis-lysogeny decisions is quite limited, especially if coupling to essential host machinery is favored. This could explain why multiple phages base their decisions on FtsH activity [4, 6, 7]. Our framework provides concrete criteria for recognizing and classifying lysis-lysogeny decision mechanisms for both well-studied and novel temperate phages.

## METHODS

### A. INCLUDING mRNA AND PROTEIN DEGRADATION TERMS

For completeness, we may also include an mRNA degradation and dilution term $-k_{\text{i}}^{\text{m}}m_{\text{i}}$ in Eq. (1). The equation can be solved with the initial condition that $m_{\text{i}}(t=0)=0$:

(5) $$m_{\text{i}}=\frac{k_{\text{i}}^{\text{tx}}g_{\text{phage}}}{k_{\text{i}}^{\text{m}}}\left(1-e^{-k_{\text{i}}^{\text{m}}t}\right).$$

Similarly, we may include a protein degradation term $-k_{\text{i}}^{\text{d}}p_{\text{i}}$ in Eq. (2). The solution is

(6) $$p_{\text{i}}=\frac{k_{\text{i}}^{\text{tr}}k_{\text{i}}^{\text{tx}}g_{\text{phage}}}{k_{\text{i}}^{\text{m}}}\left(\frac{1-e^{-k_{\text{i}}^{\text{d}}t}}{k_{\text{i}}^{\text{d}}}+\frac{e^{-k_{\text{i}}^{\text{m}}t}-e^{-k_{\text{i}}^{\text{d}}t}}{k_{\text{i}}^{\text{m}}-k_{\text{i}}^{\text{d}}}\right).$$

However, the leading order Taylor expansions for $t<1/k_{\text{i}}^{\text{m}}<1/k_{\text{i}}^{\text{d}}$, which are accurate at short times, recover the linear and quadratic dependence for mRNA and protein concentrations.

### B. DISTANCE FROM THRESHOLD FOR REPRESSED PATHWAY

Suppose $g_{1}$ and $g_{2}$ correspond to phage genome concentrations at MOIs of 1 and 2, respectively; $g_{2}=2g_{1}$. Here, we derive an expression for how far the rejected pathway’s protein concentration is from its decision threshold when the decision is reached at time $t^{*}$. For simplicity, we assume $1/k_{\text{p}}\ll t^{*}\ll 1/k_{\text{i}}^{\text{m}}\ll 1/k_{\text{lyt}}^{\text{d}}$.

First, at an MOI of 1, the decision is reached at time

(7) $$t^{*}\left(g_{1}\right)\approx \sqrt{\frac{2p_{\text{lyt}}^{*}}{k_{\text{lyt}}^{\text{tx}}k_{\text{lyt}}^{\text{tr}}g_{1}}}.$$

This implies that

(8) $$p_{\text{lyso}}\left(t^{*},g_{1}\right)\approx \frac{k_{\text{lyso}}^{\text{tr}}k_{\text{lyso}}^{\text{tx}}}{k_{\text{p}}}\sqrt{\frac{2p_{\text{lyt}}^{*}g_{1}}{k_{\text{lyt}}^{\text{tr}}k_{\text{lyt}}^{\text{tx}}}}.$$

Similarly, at an MOI of 2, we have

(9) $$t^{*}\left(g_{2}\right)\approx \frac{k_{\text{p}}p_{\text{lyso}}^{*}}{2k_{\text{lyso}}^{\mathrm{tr}}k_{\text{lyso}}^{\mathrm{tx}}g_{1}},$$

(10) $$p_{\text{lyt}}\left(t^{*},g_{2}\right)\approx \frac{k_{\text{lyt}}^{\text{tr}}k_{\text{lyt}}^{\text{tx}}k_{\text{p}}^{2}p_{\text{lyso}}^{*2}}{4g_{1}{\left(k_{\text{lyso}}^{\text{tr}}k_{\text{lyso}}^{\text{tx}}\right)}^{2}}.$$

Equations (8) and (10) can be combined to find the relation

(11) $$\frac{p_{\text{lyso}}\left(t^{*},g_{2}\right)}{p_{\text{lyso}}^{*}}\times \sqrt{\frac{p_{\text{lyt}}\left(t^{*},g_{1}\right)}{p_{\text{lyt}}^{*}}}\approx \frac{1}{\sqrt{2}}.$$

Eq. (11) describes a trade-off: if the concentration of lysogenic protein is low when the lytic decision is reached at an MOI of 1, the concentration of lytic protein will be close to the threshold when the lysogenic decision is reached at an MOI of 2. If we require $\frac{p_{\text{lyt}}\left(t^{*},g_{2}\right)}{p_{\text{lyt}}^{*}}=\frac{p_{\text{lyso}}\left(t^{*},g_{1}\right)}{p_{\text{lyso}}^{*}}$, this ratio is given by $2^{-1/3}\approx 0.8$.

### C. SCALING OF $t_{\text{x}}$ WITH $k_{\text{p}}$

The phage genome concentration $g_{\text{lyso}}$ at which the lysis-lysogeny decision switches can generally be found by solving

(12) $$\frac{p_{\text{lyt}}\left(t_{\text{x}},g_{\text{lyso}}\right)}{p_{\text{lyt}}^{*}}=\frac{p_{\text{lyso}}\left(t_{\text{x}},g_{\text{lyso}}\right)}{p_{\text{lyso}}^{*}}=1$$

for $t_{\text{x}}$ and $g_{\text{lyso}}$. Here, we show that $t_{\text{x}}=t^{*}\left(g_{\text{lyso}}\right)\sim 1/k_{\text{p}}$, where $k_{\text{p}}$ is the rate of protease degradation of the lysogeny protein. The scaling $g_{\text{lyso}}\sim k_{\text{p}}^{2}$ can then be derived as discussed in the main text.

Eq. (6) shows that even when mRNA and protein degradation and dilution are considered, $p_{\text{i}}$ only depends linearly on $g_{\text{phage}}$. This implies that the time at which the two curves cross, $t_{\text{x}}$, is independent of $g_{\text{phage}}$:

(13)

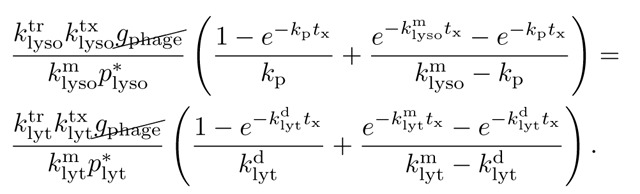



Hence, if a solution exists, $t_{\text{x}}$ only depends on the rates $k_{\text{p}}$, $k_{\text{lyt}}^{\text{d}}$, and $k_{\text{i}}^{\text{m}}$, as well as on the ratio

(14) $$R:=\frac{k_{\text{lyso}}^{\text{tx}}k_{\text{lyso}}^{\text{tr}}p_{\text{lyt}}^{*}}{k_{\text{lyt}}^{\text{tx}}k_{\text{lyt}}^{\text{tr}}p_{\text{lyso}}^{*}}.$$

Graphically, as the MOI increases, both $p_{\text{lyt}}$ and $p_{\text{lyso}}$ move upwards on a log-log plot by the same amount; hence, their crossing point moves upwards along a straight line (Fig. 1C).

By defining ${\tilde{t}}_{\text{x}}=k_{\text{p}}t_{\text{x}}$, ${\tilde{k}}_{\text{i}}^{\text{m}}=k_{\text{i}}^{\text{m}}/k_{\text{p}}$, and ${\tilde{k}}_{\text{lyt}}^{\text{d}}=k_{\text{lyt}}^{\text{d}}/k_{\text{p}}$, Eq. (13) can be written in the nondimensionalized form $F\left({\tilde{t}}_{\text{x}},{\tilde{k}}_{\text{i}}^{\text{m}},{\tilde{k}}_{\text{lyt}}^{\text{d}},R\right)=0$. When $k_{\text{p}}$ is significantly larger than $k_{\text{i}}^{\text{m}}$ and $k_{\text{lyt}}^{\text{d}}$, ${\tilde{t}}_{\text{x}}$ is approximated by the solution of $F\left({\tilde{t}}_{\text{x}},0,0,R\right)=0$. Consequently, $t_{\text{x}}\propto 1/k_{\text{p}}$, with a prefactor that only depends on R. This approximation breaks down as $k_{\text{p}}\to k_{\text{i}}^{\text{m}}$ and the crossing time $t_{\text{x}}$ approaches $1/k_{\text{i}}^{\text{m}}$. For decision mechanisms acting on timescales below $1/k_{\text{i}}^{\text{m}}$, lysogeny would then be preferred (Fig. 1B).

### D. PREFACTOR FOR EQ. (4) WHEN $1/k_{\text{p}}\ll t_{\text{x}}$

In the limit when $1/k_{\text{p}}\ll t_{\text{x}}\ll 1/k_{\text{i}}^{\text{m}}\ll 1/k_{\text{lyt}}^{\text{d}}$, the prefactor for Eq. (4) can be found analytically. We write

(15) $$\begin{array}{l} p_{\text{lyso}}=\frac{k_{\text{lyso}}^{\text{tr}}k_{\text{lyso}}^{\text{tx}}g_{\text{phage}}}{k_{\text{p}}}\left(t+\frac{1}{k_{\text{p}}}\left(e^{-k_{\text{p}}t}-1\right)\right)\\ \approx \frac{k_{\text{lyso}}^{\text{tr}}k_{\text{lyso}}^{\text{tx}}g_{\text{phage}}}{k_{\text{p}}}\left(t-\frac{1}{k_{\text{p}}}\right). \end{array}$$

The curves cross when

(16)

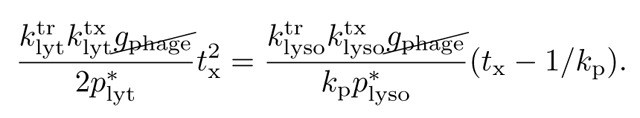



Neglecting the term $1/k_{\text{p}}$ gives a simple linear equation for $t_{\text{x}}$ in the limit $t_{\text{x}}\gg 1/k_{\text{p}}$. Here, we include the linear correction by solving the quadratic equation. Dividing both sides by $\frac{k_{\text{lyso}}^{\text{tr}}k_{\text{lyso}}^{\text{tx}}}{p_{\text{lyso}}^{*}}$, we recognize the ratio *R* (Eq. (14)), and solve for $t_{\text{x}}$:

(17) $$t_{\text{x}}=\frac{R}{k_{\text{p}}}(1\pm \sqrt{1-2/R}).$$

Note that we require R>2 for a solution to exist; graphically, this corresponds to a requirement that the $p_{\text{lyso}}$ curve is initially above the $p_{\text{lyt}}$ curve (Fig. 1C). Since we expect a larger R to correspond to a larger initial distance between the $p_{\text{i}}$ curves, $t_{\text{x}}$ should increase with *R*. We hence take the positive branch of Eq. (17).

Substituting into $p_{\text{lyso}}\left(t_{\text{x}},g_{\text{lyso}}\right)=p_{\text{lyso}}^{*}$ then gives

(18) $$p_{\text{lyso}}^{*}=\frac{k_{\text{lyso}}^{tr}k_{\text{lyso}}^{tx}g_{\text{lyso}}}{k_{\text{p}}^{2}}(R+R\sqrt{1-2/R}-1).$$

Solving for $g_{\text{lyso}}$:

(19) $$g_{\text{lyso}}=\frac{k_{\text{p}}^{2}p_{\text{lyso}}^{*}}{k_{\text{lyso}}^{tr}k_{\text{lyso}}^{tx}(R-1+R\sqrt{1-2/R})}.$$

For $1/k_{\text{p}}≲t_{\text{x}}$, $p_{\text{lyso}}(t)$ shows a transition from a linear to a quadratic temporal scaling (Fig. 1C), and the prefactor changes.

### E. SATURATION OF PROTEASE ACTIVITY

Suppose protease-mediated degradation is initially linear (Eq. (3)) but saturates to a constant level once $p_{\text{lyso}}\left(t_{\text{sat}}\right)=p_{\text{sat}}$, where $t_{\text{sat}}$ is the saturation time and $p_{\text{lyso}}(t)$ a saturation threshold concentration. The concentration curve $p_{\text{lyso}}(t)$ can now have three scaling regimes: (1) $p_{\text{lyso}}∼t^{2}$ when $t<1/k_{\text{p}}$; (2) $p_{\text{lyso}}∼t$ when $1/k_{\text{p}}<t<t_{\text{sat}}$; and (3) $p_{\text{lyso}}∼t^{2}$ once protease activity saturates for $t>t_{\text{sat}}$. At higher MOIs, $p_{\text{sat}}$ is reached faster, and the duration of regime (2) decreases.

If $p_{\text{sat}}<p_{\text{lyso}}^{*}$, the lytic pathway is destabilized: once $p_{\text{lyso}}\left(1/k_{\text{p}}\right)>p_{\text{sat}}$, regime II ceases to exist, and lysogeny is preferred (Fig. 1B). Furthermore, if a lysis decision is reached at time $t^{*}$, $p_{\text{lyso}}\left(t^{*}\right)$ is higher than in the absence of protease activity saturation.

If $p_{\text{sat}}≳p_{\text{lyso}}^{*}$, the lysogenic pathway is stabilized. In this case, protease activity saturation does not significantly affect $p_{\text{i}}(t)$ for $t<t^{*}$. Hence, the same MOI-based decision is reached. If the lytic decision is reached and $p_{\text{lyso}}$ is degraded before reaching $p_{\text{sat}}$, the lytic pathway remains stable. By contrast, if a lysogeny decision is made, $p_{\text{lyso}}$ reaches saturation levels, enhancing its net accumulation after the decision.

## References

[1] Kourilsky P., enLysogenization by bacteriophage lambda, Molecular and General Genetics MGG 122, 183 (1973). 4573866 10.1007/BF00435190

[2] Oppenheim A. B., Kobiler O., Stavans J., Court D. L., and Adhya S., enSwitches in Bacteriophage Lambda Development, Annual Review of Genetics 39, 409 (2005). 10.1146/annurev.genet.39.073003.113656 16285866

[3] Casjens S. R. and Hendrix R. W., Bacteriophage lambda: Early pioneer and still relevant, Virology 60th Anniversary Issue, 479-480, 310 (2015). 10.1016/j.virol.2015.02.010 PMC4424060 25742714

[4] Herman C., Ogura T., Tomoyasu T., Hiraga S., Akiyama Y., Ito K., Thomas R., D’Ari R., and Bouloc P., Cell growth and lambda phage development controlled by the same essential Escherichia coli gene, ftsH/hflB., Proceedings of the National Academy of Sciences 90, 10861 (1993). 10.1073/pnas.90.22.10861 PMC47878 8248182

[5] Shotland Y., Koby S., Teff D., Mansur N., Oren D. A., Tatematsu K., Tomoyasu T., Kessel M., Bukau B., Ogura T., and Oppenheim A. B., engProteolysis of the phage lambda CII regulatory protein by FtsH (HflB) of Escherichia coli, Molecular Microbiology 24, 1303 (1997). 9218777 10.1046/j.1365-2958.1997.4231796.x

[6] Broussard G. W., Oldfield L. M., Villanueva V. M., Lunt B. L., Shine E. E., and Hatfull G. F., EnglishIntegration-Dependent Bacteriophage Immunity Provides Insights into the Evolution of Genetic Switches, Molecular Cell 49, 237 (2013). 23246436 10.1016/j.molcel.2012.11.012 PMC3557535

[7] Murchland I. M., Ahlgren-Berg A., Pietsch J. M. J., Isabel A., Dodd I., and Shearwin K. E., Instability of CII is needed for efficient switching between lytic and lysogenic development in bacteriophage 186, Nucleic Acids Research 48, 12030 (2020). 33211866 10.1093/nar/gkaa1065 PMC7708051

[8] Weitz J. S., Mileyko Y., Joh R. I., and Voit E. O., EnglishCollective Decision Making in Bacterial Viruses, Biophysical Journal 95, 2673 (2008). 18567629 10.1529/biophysj.108.133694 PMC2527279

[9] Robb M. L. and Shahrezaei V., enStochastic Cellular Fate Decision Making by Multiple Infecting Lambda Phage, PLOS ONE 9, e103636 (2014). 10.1371/journal.pone.0103636 PMC4126663 25105971

[10] Yao T., Coleman S., Nguyen T. V. P., Golding I., and Igoshin O. A., Bacteriophage self-counting in the presence of viral replication, Proceedings of the National Academy of Sciences 118, e2104163118 (2021). 10.1073/pnas.2104163118 PMC8713767 34916284

[11] Gupta M., Johnson A. N. T., Cruz E. R., Costa E. J., Guest R. L., Li S. H.-J., Hart E. M., Nguyen T., Stadlmeier M., Bratton B. P., Silhavy T. J., Wingreen N. S., Gitai Z., and Wühr M., enGlobal protein turnover quantification in Escherichia coli reveals cytoplasmic recycling under nitrogen limitation, Nature Communications 15, 5890 (2024). 10.1038/s41467-024-49920-8 PMC11246515 39003262

[12] Herman C., Thévenet D., D’Ari R., and Bouloc P., The HflB protease of Escherichia coli degrades its inhibitor lambda cIII., Journal of Bacteriology 179, 358 (1997). 8990286 10.1128/jb.179.2.358-363.1997 PMC178704

[13] Obuchowski M., Shotland Y., Koby S., Giladi H., Gabig M., Wegrzyn G., and Oppenheim A. B., Stability of CII is a key element in the cold stress response of bacteriophage lambda infection, Journal of Bacteriology 179, 5987 (1997). 9324241 10.1128/jb.179.19.5987-5991.1997 PMC179497

[14] Słomińska M., Neubauer P., and Wegrzyn G., Regulation of Bacteriophage Development by Guanosine 5-Diphosphate-3-diphosphate, Virology 262, 431 (1999). 10502521 10.1006/viro.1999.9907

[15] Geng Y., Nguyen T. V. P., Homaee E., and Golding I., enUsing bacterial population dynamics to count phages and their lysogens, Nature Communications 15, 7814 (2024). 10.1038/s41467-024-51913-6 PMC11379933 39242585

